# Intravenous Iron Therapy in Patients Admitted With Acute Heart Failure and Iron Deficiency: A Systematic Review and Meta-Analysis

**DOI:** 10.7759/cureus.88989

**Published:** 2025-07-29

**Authors:** Victor Lopez, Melissa Chacón, María Jesús Arias, Anthony Rojas, Jessica Arias, Paula Vanegas, Fiorella Apuy Rodríguez, Manuel E Saenz

**Affiliations:** 1 Hospital Medicine, Sinai Hospital of Baltimore, Baltimore, USA; 2 Faculty of Medicine, Universidad de Costa Rica, San José, CRI; 3 Faculty of Medicine, Universidad Hispanoamericana, San José, CRI; 4 Interventional Cardiology, Centro Cardiovascular SM, San José, CRI

**Keywords:** acute heart failure, intravenous iron, iron deficiency, mortality, rehospitalization

## Abstract

The role of intravenous (IV) iron in chronic heart failure (HF) has been well studied, becoming a class IA recommendation. However, its role in acute heart failure (AHF) is less well-known. Multiple studies, including randomized controlled trials (RCTs), have been published; however, their clinical benefit remains controversial. We aim to provide enough evidence to support decision-making in this clinical scenario. We performed a systematic review and meta-analysis of IV iron in patients admitted with AHF and iron deficiency (ID). PubMed, Embase, Scopus, and Cochrane databases were searched for trials published up to July 1, 2024. Risk ratios (RRs) and mean differences (MDs) with 95% confidence intervals (CIs) were pooled across trials. Outcomes included HF and all-cause re-hospitalization, all-cause mortality, and mean change in hemoglobin levels. Of the 362 database results, three RCTs, six observational studies, and 3,588 patients were included. In total, 1,622 (45.2%) patients received IV iron. Re-hospitalization for HF (RR = 0.96; 95% CI = 0.76-1.21; p = 0.74; I² = 74%) showed a downward trend, but this was not statistically significant. Neither was all-cause rehospitalization (RR = 1.03; 95% CI = 0.90-1.19; p = 0.64; I² = 3%) nor all-cause mortality (RR = 1.00; 95% CI = 0.81-1.24; p = 0.87; I² = 0%). A statistically significant mean change in the hemoglobin levels (MD = 0.80; 95% CI = 0.33-1.27; p = 0.0003; I² = 88%) was documented between both groups. In patients with AHF and ID, treatment with IV iron improves hemoglobin levels. Yet, this improvement does not appear to have a significant impact on rehospitalization or all-cause mortality rates. Larger RCTs are needed to further study its effect on clinical outcomes.

## Introduction and background

Iron deficiency (ID) affects approximately 50% of patients with chronic heart failure (CHF), regardless of anemia status, and up to 80% of those with acute heart failure (AHF) [1]. ID is associated with an increased risk of hospitalization, reduced exercise capacity, worsening heart failure (HF) symptoms, decreased quality of life, and other complications [2,3]. Numerous studies have demonstrated that treating ID in CHF patients reduces HF-related hospitalizations and improves symptoms and functional status [4]. As a result, the 2021 European Society of Cardiology (ESC) guidelines on AHF and CHF recommend intravenous (IV) iron therapy as a Class IA treatment for symptomatic patients with heart failure with reduced ejection fraction (HFrEF) or mildly reduced ejection fraction (HFmrEF) and concomitant ID to improve quality of life [1].

In contrast, the role of IV iron therapy in AHF remains unclear. The diagnosis of ID in this setting is more complex, as ferritin, an acute-phase reactant, may be elevated due to inflammation, potentially masking true ID [5]. Although several recent studies, including randomized controlled trials (RCTs), have explored the efficacy of IV iron in AHF patients, its clinical benefits remain controversial.

This work was previously presented as a meeting abstract at the 2024 AMA Research Challenge on November 8, 2024. Our objective was to assess whether IV iron therapy improves mortality, rehospitalization, and hematologic parameters in patients hospitalized with AHF and ID when used alongside standard guideline-directed therapy.

## Review

Methodology

This systematic review and meta-analysis was conducted using the Cochrane Collaboration Handbook for Systematic Reviews of Interventions [6] and the Preferred Reporting Items for Systematic Reviews and Meta-Analyses (PRISMA) guidelines [7]. The study protocol was prospectively registered in the International Prospective Register of Systematic Reviews (PROSPERO) (registration number: CRD420251089581). Studies were excluded if they lacked a comparison group, did not report outcomes of interest, or included patients without a confirmed diagnosis of AHF.

Search Strategy

We systematically searched PubMed, Embase, Scopus, and Cochrane databases for trials published from inception up to December 31, 2024. The following key terms were used: “acute heart failure,” “AHF,” “acute decompensated heart failure,” “ADHF,” “iron deficiency,” “intravenous iron,” “IV iron,” “ferric carboxymaltose,” “iron sucrose,” “oral iron,” and “ferrous sulfate.”

Eligibility Criteria

We included RCTs and therapeutic and observational studies that involved patients diagnosed with AHF and ID anemia, who received IV iron and standard care versus standard care alone and reported any of our outcomes of interest, namely, all-cause mortality, HF and all-cause rehospitalization, and mean change in hemoglobin levels. ID was defined as: (1) ferritin level <100 μg/L, (2) ferritin level between 100 and 300 μg /L with a transferrin saturation <20%, and (3) transferrin saturation <20%.

Data Extraction and Statistical Analysis

Two authors (V.L. and M.C.) independently extracted the baseline characteristics and outcome data according to the predetermined criteria for the literature search, data extraction, and quality assessment. Any discrepancies were resolved through consensus. Risk ratios (RRs) and mean differences (MDs) with 95% confidence intervals (CIs) were calculated to assess treatment effects for binary and continuous outcomes, respectively, with statistical significance defined as a p-value <0.05. Results were visually presented using a forest plot. Study heterogeneity was evaluated using the Cochran Q test and the I² statistic. We applied Cochrane’s predefined thresholds for heterogeneity interpretation: 0-40% (minimal), 30-60% (moderate), 50-90% (substantial), and 75-100% (considerable). All statistical analyses were performed using RStudio version 4.2.2 (R Foundation for Statistical Computing, Vienna, Austria).

Quality Assessment

RCTs were assessed using the Cochrane Risk of Bias 2 (RoB 2) tool, which evaluates bias across five key domains, including the randomization process, deviations from intended interventions, missing outcome data, outcome measurement, and selective reporting [17]. Non-randomized studies were evaluated using the Risk of Bias in Non-Randomized Studies of Interventions (ROBINS-I) tool, which assesses bias due to confounding, participant selection, intervention classification, deviations from intended interventions, missing data, outcome measurement, and selective reporting [18].

Results

Study Selection and Baseline Characteristics

**Figure 1:**
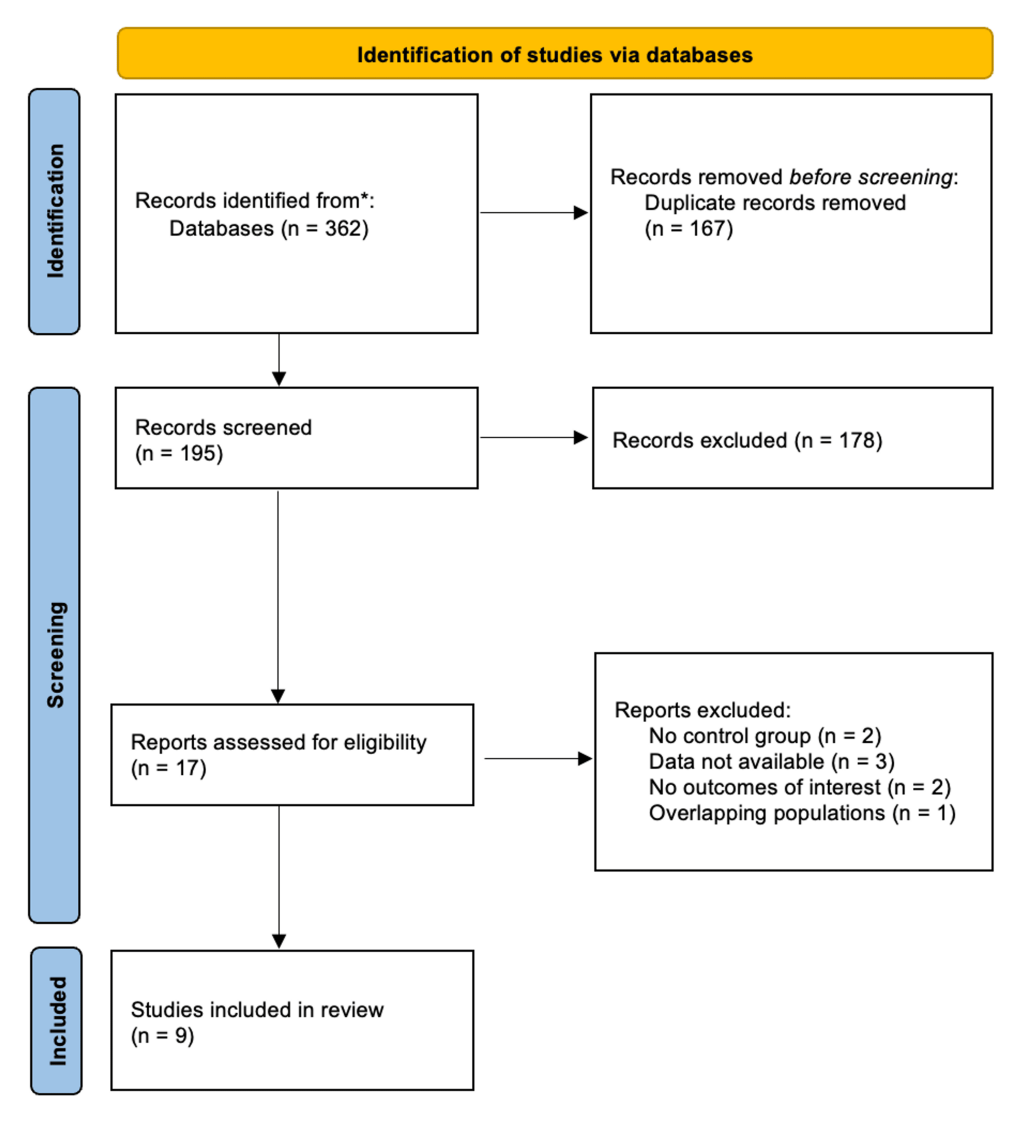
Preferred Reporting Items for Systematic Reviews and Meta-Analyses (PRISMA) flow diagram showing study screening and selection.

As shown in Figure 1, our initial database search yielded 362 records. After removing 167 duplicates and excluding 177 studies based on title and abstract screening, 17 articles were assessed in full against our inclusion criteria. Following detailed evaluation, nine studies, comprising three RCTs and six observational studies, were deemed eligible for inclusion in this systematic review and meta-analysis. These studies included a total of 3,588 patients, of whom 1,622 (45.2%) received IV iron therapy. The patient population was predominantly male (1,884, 53.4%), with a mean age of 72.22 years. Among those who received IV iron, 1,332 (81.87%) patients were treated with ferric carboxymaltose. Study characteristics are summarized in Table 1.

**Table 1:** Design and characteristics of the studies included in the meta-analysis. ADHF: acute decompensated heart failure; AHF: acute heart failure; CT: control; FCM: ferric carboxymaltose; Hb: hemoglobin; HF: heart failure; ID: iron deficiency; IDA: iron deficiency anemia; IV: intravenous; IT: iron therapy; LVEF: low ventricular ejection fraction; NA: not available; RCT: randomized controlled trial; SFGC: sodium ferric gluconate complex; NTproBNP: N-terminal pro-B-type natriuretic peptide; TSAT: transferrin saturation

Study	Study design	Patients IT/CT (n)	Males IT/CT (%)	Age IT/CT (years)	Follow-up	Patient population	ID definition	IV iron therapy	Control group	Outcomes available
Ponikowski et al. (AFFIRM-AHF) [8]	RCT	558/550	56/55	71.2/70.9	52 weeks	>18 years old, hospitalized for AHF, treated with furosemide 40 mg (or equivalent), LVEF <50%, ID	Ferritin <100 ng/mL or 100–299 ng/mL + TSAT <20	FCM	Placebo	HF rehospitalization. Change in Hb level
Youssef al. [9]	RCT	40/20	NA	NA	3 months	ADHF, NYHA class III-IV, LVEF <40%, anemia, and ID	Ferritin <100 μg/L or 100–299 μg/L + TSAT <20%	Iron sucrose	Placebo (IV saline)	All-cause rehospitalization. All-cause mortality. Change in Hb level
Yeo et al. (PRACTICE-ASIA-HF) [10]	RCT	24/25	75/80	61.1/64	12 weeks	>21 years old, ADHF, IDA, able to complete a 6-minute walk test	Ferritin <300 ng/mL + TSAT <20%	FCM	Placebo (IV saline)	HF rehospitalization
Jacob et al. (AHF-ID) [11]	Prospective	158/33	38/27.3	84.3/84.6	12 months	AHF and ID	Ferritin <100 μg/L or 100–299 μg/L + TSAT<20%	FCM	NA	HF rehospitalization. All-cause mortality
Mistry et al. [12]	Prospective	84/205	69.9/47.9	61.9/66.4	12 months	AHF and ID	Ferritin <100 ng/mL or 100–300 ng/mL + TSAT <20%	Iron sucrose	NA	HF rehospitalization. All-cause rehospitalization. All-cause mortality
Kaminsky et al. [13]	Retrospective	44/128	48/62	61/66	30 days	>18 years old, with AHF, ID	TSAT <20%	Iron dextran or sucrose	NA	All-cause rehospitalization
Capone et al. [14]	Retrospective	52/15	59.6/86.6	83/82.6	500 days	ADHF and ID	Ferritin <100 μg/L or 100–99 μg/L + TSAT < 20%	FCM	NA	All-cause mortality. Change in Hb level
Borreda et al. [15]	Retrospective	122/718	59.8/51.3	71.66/74.85	400 days	>18 years old, primary diagnosis of ADHF (NTproBNP >300), IDA, treated with loop diuretics	Ferritin <100 μg /L or 100–400 μg/L + TSAT<20%	SFGC	NA	HF rehospitalization. All-cause mortality
López-Vilella et al. [16]	Retrospective	540/272	54.4/52.2	76.65/75.45	6 months	ADHF and ID	Ferritin <100 μg/L or 100–300 μg/L + TSAT <20%	FCM	NA	HF rehospitalization. All-cause mortality

Qualitative Synthesis

This meta-analysis included a total of nine studies, with six observational cohort studies and three RCTs. Among the observational studies, Borreda et al. [15], López-Vilella et al. [16], Capone et al. [14], Kaminsky et al. [13], and Mistry et al. [12] employed retrospective designs using hospital databases or medical records, while the AHF-ID study was a prospective, multicenter cohort. All observational cohorts focused on patients hospitalized for acute decompensated HF with concurrent ID. This was defined similarly across studies using ferritin and transferrin saturation thresholds. However, the decision to administer IV iron was non-randomized and frequently left to the physician’s discretion, which introduces potential confounding despite attempts at statistical adjustment in several studies.

The three RCTs, AFFIRM-AHF [8], PRACTICE-ASIA-HF [10], and Youssef et al. [9], used placebo-controlled designs to compare IV iron (ferric carboxymaltose or iron sucrose) with standard care. AFFIRM-AHF was the largest and most methodologically rigorous trial, employing stratified randomization, masking protocols to preserve blinding despite the visible nature of the intervention, and adjustment for key prognostic factors in the statistical analysis. PRACTICE-ASIA-HF and Youssef et al. were smaller in scale, with the latter applying a 2:1 randomization ratio and shorter follow-up duration.

Across all studies, definitions of ID and anemia were consistent, aligning with international HF guidelines. Despite differences in design, follow-up duration, and analytical methods, all studies shared a common goal, namely, to assess the safety and efficacy of IV iron repletion during or shortly after hospitalization for acute decompensated HF in iron-deficient patients. These shared characteristics enhance the relevance of pooled estimates while also highlighting the methodological heterogeneity that must be considered when interpreting the findings of this meta-analysis.

Pooled Analysis of all Studies

**Figure 2:**
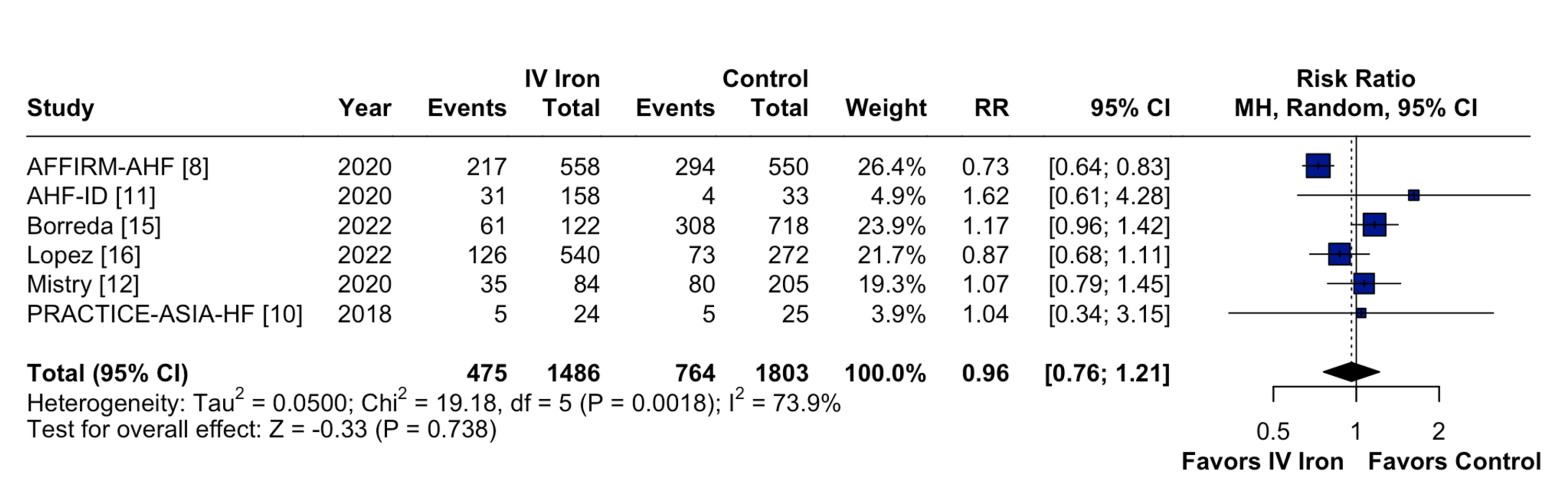
No statistically significant difference in heart failure rehospitalizations.

**Figure 3:**
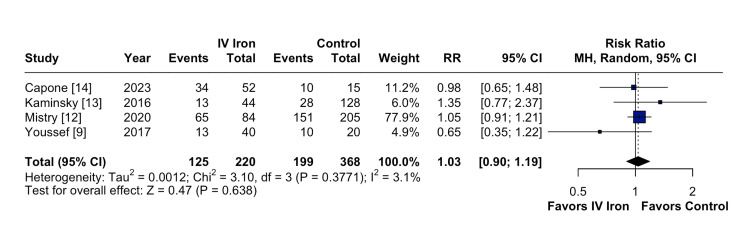
No statistically significant difference in all-cause rehospitalizations.

**Figure 4:**
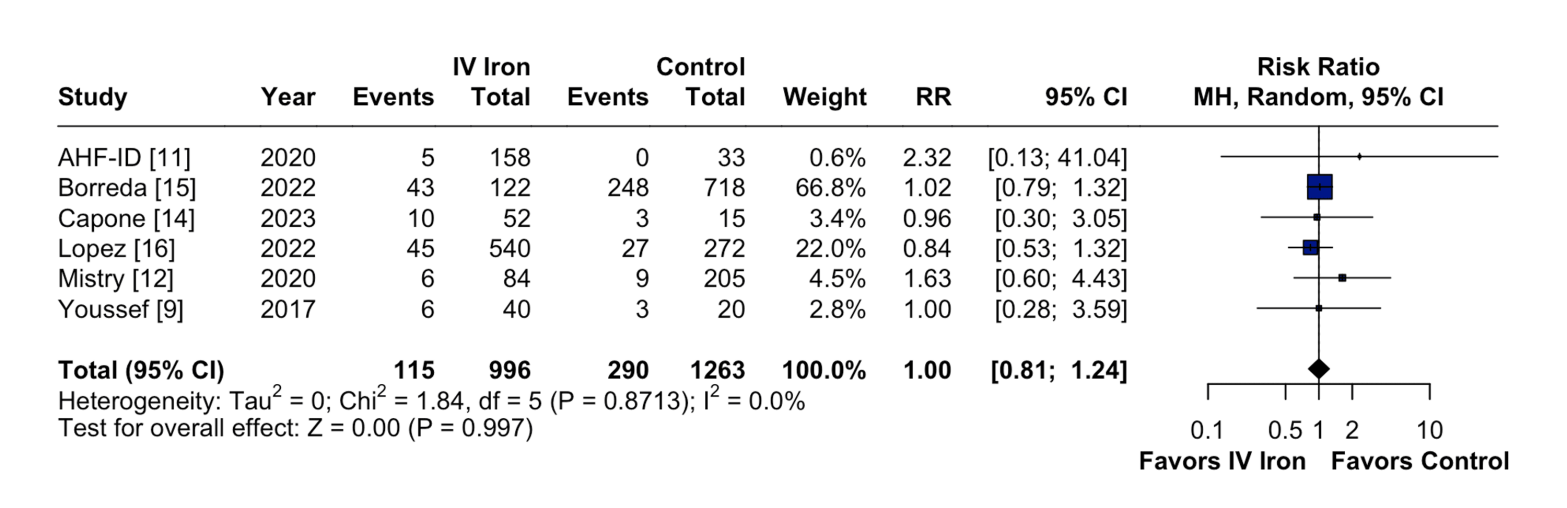
No statistically significant difference in all-cause mortality.

**Figure 5:**
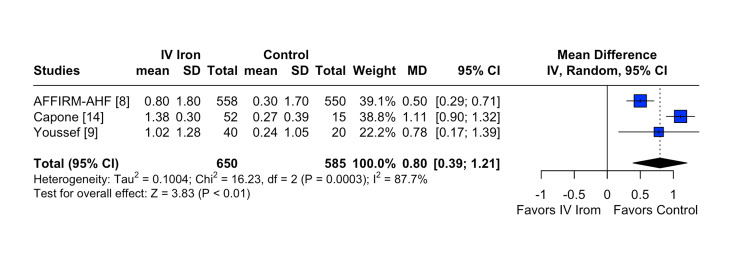
Statistically significant change in hemoglobin levels.

The pooled analysis indicated that IV iron therapy was not associated with a statistically significant reduction in hospital readmissions. Specifically, HF rehospitalizations (Figure 2) showed an RR of 0.96 (95% CI = 0.76-1.21; p = 0.74; I² = 74%), and all-cause rehospitalizations (Figure 3) had an RR of 1.03 (95% CI = 0.90-1.19; p = 0.64; I² = 3%), both reflecting non-significant effects. Similarly, all-cause mortality (Figure 4) did not differ between groups (RR = 1.00; 95% CI = 0.81-1.24; p = 0.87; I² = 0%). In contrast, IV iron therapy was associated with a statistically significant increase in hemoglobin levels (MD = 0.80 g/dL; 95% CI = 0.33-1.27; p = 0.0003; I² = 88%) (Figure 5).

Risk of Bias in Included Studies and GRADE Assessment

Quality assessment of the included studies was performed independently by M.C. and F.A., with any discrepancies resolved through discussion and consensus. All observational studies were evaluated using the ROBINS-I tool and were judged to have a moderate overall risk of bias. This rating was primarily driven by concerns regarding confounding and, in some cases, the selection of participants or reporting biases (Table 2).

**Table 2:** Risk of Bias in Non-randomized Studies of Interventions (ROBINS-I) tool for risk of bias assessment.

Study	Bias due to confounding	Bias in the selection of participants into the study	Bias in the classification of interventions	Bias due to deviations from intended interventions	Bias due to missing data	Bias in the measurement of outcomes	Bias in the selection of the reported result	Overall bias
Borreda et al. (2022) [15]	Moderate	Low	Low	Low	Moderate	Low	Low	Moderate
López-Vilella et al. (2022) [16]	Moderate	Low	Low	Low	Moderate	Low	Moderate	Moderate
Capone et al. (2023) [14]	Moderate	Moderate	Low	Moderate	Low	Moderate	Moderate	Moderate
Kaminsky et al. (2016) [13]	Moderate	Low	Low	Low	Low	Moderate	Moderate	Moderate
Mistry et al. (2019) [12]	Moderate	Low	Low	Low	Moderate	Low	Moderate	Moderate
AHF-ID (2020) [11]	Moderate	Moderate	Low	Moderate	Moderate	Moderate	Moderate	Moderate

The three RCTs were assessed using the RoB-2 tool. Among them, the AFFIRM-AHF trial demonstrated low risk of bias across all domains (Table 3). The PRACTICE-ASIA-HF trial was deemed to have some concerns due to missing outcome data. The study by Youssef et al. also showed some concerns across multiple domains, including the randomization process, deviations from intended interventions, and outcome assessment.

**Table 3:** Risk of bias summary for randomized studies (Cochrane Risk of Bias 2 tool).

Study	Bias from the randomization process	Bias due to deviations from intended interventions	Bias due to missing outcome data	Bias in the measurement of the outcomes	Bias in the selection of the reported result	Overall risk of bias
AFFIRM-AHF (2020) [8]	Low	Low	Low	Low	Low	Low
PRACTICE-ASIA-HF (2018) [10]	Low	Low	Some concerns	Low	Low	Some concerns
Youssef et al. (2017) [9]	Some concerns	Some concerns	Some concerns	Some concerns	Some concerns	Some concerns

Finally, we assessed the certainty of the evidence using the GRADE approach for each outcome. Ratings considered factors such as study design, risk of bias, inconsistency, indirectness, imprecision, and publication bias (Table 4).

**Table 4:** GRADE assessment results. ^a^: Outcome carried out with studies with a moderate risk of bias. ^b^: High heterogeneity. ^c^: Wide confidence interval. GRADE Working Group grades of evidence. High certainty: We are very confident that the true effect lies close to that of the estimate of the effect. Moderate certainty: We are moderately confident in the effect estimate. The true effect is likely to be close to the estimate of the effect, but there is a possibility that it is substantially different. Low certainty: Our confidence in the effect estimate is limited. The true effect may be substantially different from the estimate of the effect. Very low certainty: We have very little confidence in the effect estimate. The true effect is likely to be substantially different from the estimated effect. IV: intravenous; IDA: iron deficiency anemia; AHF: acute heart failure; RR: relative risk; CI: confidence interval

IV iron compared to standard care for IDA in AHF					
Patient or population: IDA in AHF intervention: IV iron comparison: standard care					
Outcomes	Number of participants (studies) Follow-up	Certainty of the evidence (GRADE)	Relative effect (95% CI)	Anticipated absolute effects	
				Risk with standard care	Risk difference with IV iron
Heart failure hospitalizations	3,289 (6 non-randomized studies)	⨁⨁◯◯ Low^a,b,c^	RR = 0.96 (0.76 to 1.21)	424 per 1,000	17 fewer per 1,000 (102 fewer to 89 more)
All-cause mortality	2,259 (6 non-randomized studies)	⨁⨁⨁◯ Moderate^a,c^	RR = 1.00 (0.81 to 1.24)	230 per 1,000	0 fewer per 1,000 (44 fewer to 55 more)
All-cause rehospitalization	588 (4 non-randomized studies)	⨁⨁⨁◯ Moderate^a,c^	RR = 1.03 (0.90 to 1.19)	541 per 1,000	16 more per 1,000 (54 fewer to 103 more)

Sensitivity Analysis

**Figure 6:**
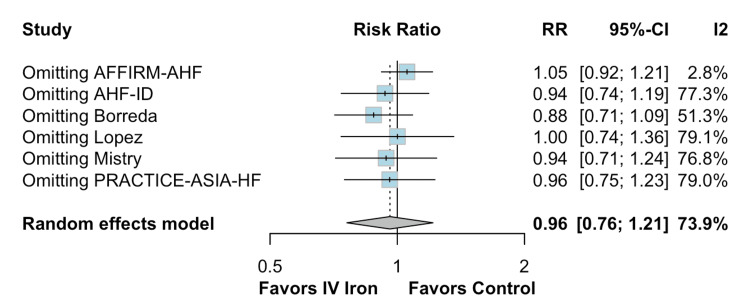
Leave-one-out sensitivity analysis for heart failure rehospitalizations.

A leave-one-out sensitivity analysis was performed to evaluate the robustness of the findings. Regarding HF rehospitalizations (Figure 6), the pooled RR was 0.96 (95% CI = 0.76-1.21), with substantial heterogeneity (I² = 73.9%). Excluding individual studies did not affect the overall estimate, which ranged from 0.88 to 1.05. Notably, omitting the AFFIRM-AHF trial resulted in the largest shift in effect size (RR = 1.05; 95% CI = 0.92-1.21) and markedly reduced heterogeneity to 2.8%, suggesting that this study contributed significantly to the between-study variance.

**Figure 7:**
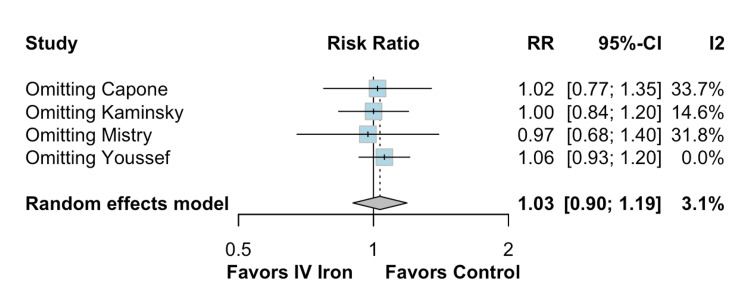
Leave-one-out sensitivity analysis for all-cause rehospitalizations.

In the case of all-cause rehospitalizations (Figure 7), the RR remained stable at 1.03 (95% CI = 0.90-1.19), with minimal heterogeneity (I² = 3.1%). Across all iterations, the estimates showed little variability (range = 0.97-1.06), and the removal of individual studies such as Capone et al., Kaminsky et al., Mistry et al., or Youssef et al. did not significantly alter the overall findings or heterogeneity. This consistency underscores the robustness of the result.

**Figure 8:**
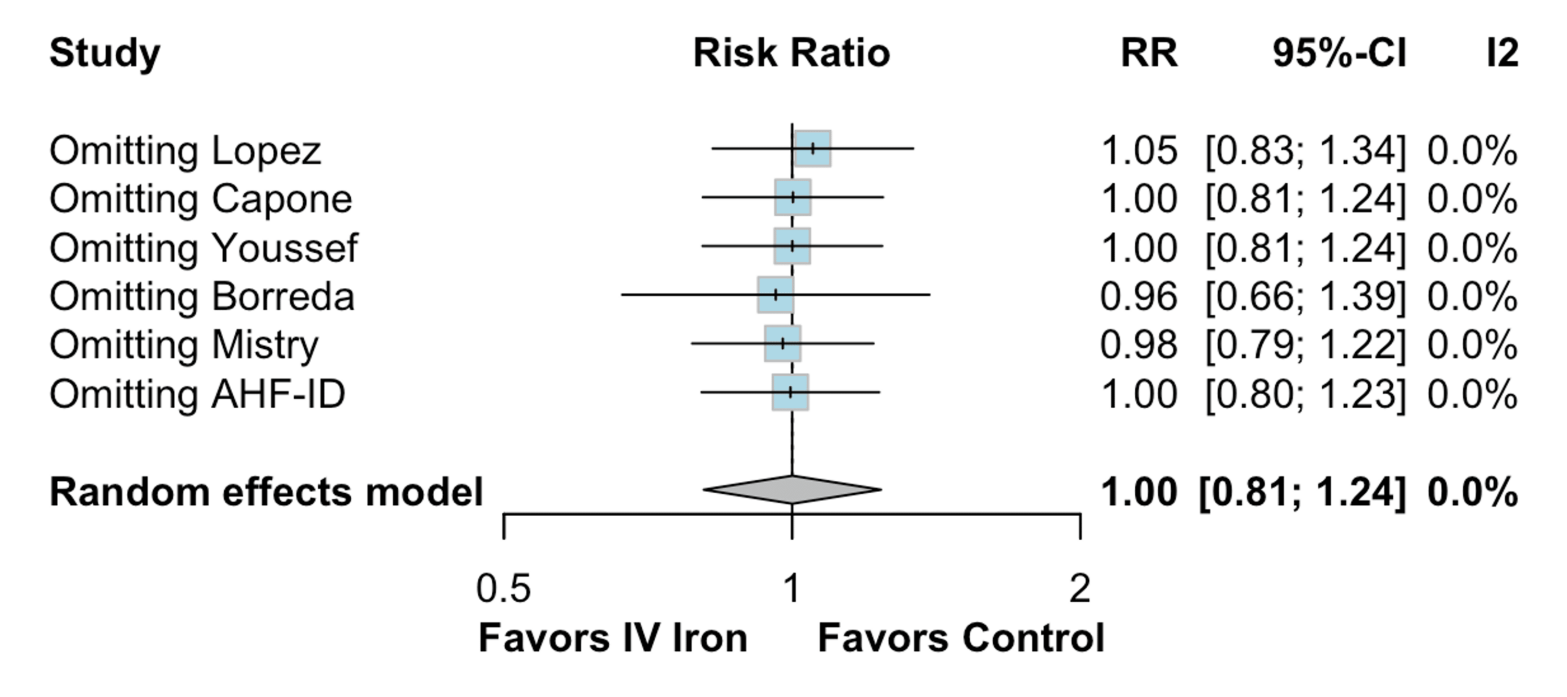
Leave-one-out sensitivity analysis for all-cause mortality.

The findings for all-cause mortality (Figure 8) were equally stable, with a pooled RR of 1.00 (95% CI = 0.81-1.24) and no observed heterogeneity (I² = 0.0%). The exclusion of any single study, including Lopez et al., Capone et al., Youssef et al., Borreda et al., Mistry et al., or AHF-ID, had minimal impact on the effect size, which varied narrowly between 0.96 and 1.05. These results highlight the reliability of the mortality outcome.

**Figure 9:**
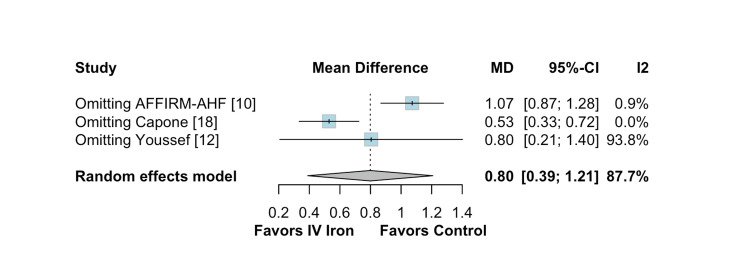
Leave-one-out sensitivity analysis for hemoglobin change.

In contrast, the analysis of hemoglobin change (Figure 9) revealed greater sensitivity to individual studies. The pooled MD was 0.80 g/dL (95% CI = 0.39-1.21), accompanied by high heterogeneity (I² = 87.7%). Omitting AFFIRM-AHF increased the MD to 1.07 (95% CI = 0.87-1.28) and substantially reduced heterogeneity to 0.9%, whereas omitting Capone et al. reduced the effect size to 0.53 (95% CI = 0.33-0.72), with an I² of 0.0%. These results indicate that specific studies, particularly AFFIRM-AHF and Capone et al., exerted a notable influence on the overall estimate and contributed meaningfully to heterogeneity.

Discussion

In this meta-analysis, we explored the impact of IV iron therapy in patients with AHF and ID, providing insights into its clinical implications. After an extensive review, 362 studies were initially identified, and a total of nine studies met the inclusion criteria, comprising six observational studies and three RCTs. The key findings of our analysis include: (1) IV iron therapy led to a statistically significant increase in hemoglobin levels compared to the control group, reinforcing its potential to correct ID. (2) Despite this hematologic improvement, IV iron therapy did not significantly reduce rehospitalization rates in AHF, whether specific to HF or all causes. These findings indicate that hemoglobin elevation alone is insufficient to drive clinical benefits, highlighting the necessity of patient stratification for optimized IV iron therapy. (3) IV iron therapy did not influence all-cause mortality, underscoring the necessity of larger RCTs to evaluate its long-term impact on survival outcomes. (4) Considerable heterogeneity was observed, particularly in hemoglobin level changes (I² = 88%), which may be due to differences in IV iron formulations, dosing strategies, and treatment duration across studies. These findings warrant further exploration into both the biological plausibility and clinical application of IV iron in AHF.

Several factors may explain the absence of both mortality and rehospitalization benefits despite the observed hematologic improvement. First, most included studies had relatively short follow-up durations, potentially limiting the detection of long-term clinical effects. Additionally, while IV iron has been shown to improve oxygen transport and mitochondrial function, these effects may not be sufficient to impact outcomes such as arrhythmias, fluid overload, or myocardial remodeling in the short term. Furthermore, variations in patient characteristics (e.g., left ventricular ejection fraction (LVEF) phenotype, comorbidities), iron formulations, dosing protocols, and timing of administration likely contributed to clinical heterogeneity and may have diluted any potential impact on rehospitalization rates. These limitations underscore the need for more targeted trials with longer follow-up, standardized intervention protocols, and subgroup analyses to identify patients most likely to benefit from IV iron therapy in the acute setting.

Future research must establish standardized protocols to elucidate the clinical impact and reduce heterogeneity in management approaches following IV iron administration. The results of this meta-analysis align with previous studies on IV iron therapy in AHF, supporting its role in hematologic improvement while leaving uncertainty regarding its impact on clinical outcomes [8,9,15]. Similar to trials such as AFFIRM-AHF and PRACTICE-ASIA-HF, our findings confirm that while IV iron effectively raises hemoglobin levels, it does not significantly reduce rehospitalization or mortality [8,10]. The consistency with prior studies indicates that IV iron repletion alone fails to produce significant short-term clinical improvements, underscoring the importance of further investigations into long-term treatment strategies [8,11,12]. In CHF, the primary focus is on long-term functional improvement, whereas in AHF, the immediate priority is hemodynamic stabilization and acute decongestion [8,9,15,16]. These differences stem from distinct pathophysiological mechanisms of these conditions, which can influence how IV iron therapy impacts clinical outcomes. Understanding the pathophysiological rationale behind iron therapy in HF may help explain the disconnect between hematologic improvements and clinical outcomes.

Mechanistically, iron plays a central role in myocardial energetics. In states of ID, mitochondrial function is impaired, oxidative phosphorylation is reduced, and ATP production is compromised, leading to impaired cardiac contractility and reduced exercise tolerance. IV iron repletion may restore these processes by improving oxygen delivery, enhancing mitochondrial efficiency, reducing oxidative stress, and promoting myocardial contractility [8,16]. Additionally, IV iron may help reverse the inhibitory effects of elevated hepcidin levels, commonly seen in inflammatory states such as AHF, which disrupt iron metabolism and cellular uptake [13,14,16]. These theoretical benefits provide a strong biological rationale for its use, although they have not yet consistently translated into improved short-term clinical outcomes in acute settings.

Despite these advantages, IV iron has not consistently influenced hemodynamic markers such as N-terminal pro-B-type natriuretic peptide, which may result from its limited effect on congestion and volume overload [16]. Reliable biomarkers are needed to predict response, refine patient selection, and optimize timely interventions. IV iron therapy has demonstrated a reduction in rehospitalization in CHF, particularly in HFrEF. However, in AHF, this effect has not been consistent across LVEF subgroups [8,16]. Some studies have reported greater increases in ferritin and transferrin saturation in heart failure with preserved ejection fraction (HFpEF) patients compared to HFrEF, with no comparable improvements in ejection fraction, yet no clear impact on rehospitalization rates [8,16]. This suggests that the clinical benefits of IV iron may be influenced by underlying HF phenotypes and the acute or chronic nature of the condition. This emphasizes the importance of further research to determine whether stratified treatment approaches can maximize clinical benefits. Future investigations should also explore the potential advantages of maintenance IV iron therapy, as a single administration may not provide sustained benefits [14]. Demonstrated safety profiles of IV iron support the feasibility of long-term strategies to optimize its use in clinical practice [8,11,13,19].

Limitations

While our findings offer important insights, some limitations must be acknowledged. First, the inclusion of both observational and randomized studies introduces potential sources of bias. Although we applied random-effects models and assessed heterogeneity using I² statistics, differences in study design, iron formulations, follow-up duration, and outcome definitions could affect the robustness of the pooled estimates. Second, some studies enrolled heterogeneous HF populations without stratifying outcomes by LVEF. As a result, patients with HFpEF may have been included in the analysis. Given the differing pathophysiology between HFpEF and HFrEF, particularly concerning myocardial energetics and response to iron repletion, this may have influenced treatment effects and contributed to variability across studies. Third, subgroup analyses were not possible due to incomplete data on baseline LVEF, iron indices, or comorbidities, limiting our ability to assess differential treatment effects. Despite these limitations, this study provides a comprehensive synthesis of available evidence and highlights the need for future trials with standardized protocols, stratified analyses, and longer-term follow-up to refine patient selection and optimize clinical impact.

## Conclusions

This meta-analysis, encompassing 3,588 patients, found that IV iron therapy in individuals hospitalized for AHF with ID anemia significantly increased hemoglobin levels. However, it did not result in a statistically significant reduction in rehospitalization rates or all-cause mortality. Our findings are consistent with the current 2021 ESC guidelines, which provide a Class IA recommendation for IV iron in CHF but make no formal recommendation for its use in AHF due to limited evidence. The absence of mortality or rehospitalization benefit in our pooled analysis reinforces this cautious position. This highlights the need for future studies using standardized treatment protocols and unified ID definitions, with adequate follow-up to evaluate long-term effects beyond hemoglobin correction. Such research should also examine the role of patient phenotypes, such as HF subtype or baseline iron indices, in guiding personalized treatment strategies and informing future updates to practice guidelines.
